# Impedance self-matching ultra-narrow linewidth fiber resonator by use of a tunable *π*-phase-shifted FBG

**DOI:** 10.1038/s41598-017-02112-5

**Published:** 2017-05-15

**Authors:** Mingyong Jing, Bo Yu, Jianyong Hu, Huifang Hou, Guofeng Zhang, Liantuan Xiao, Suotang Jia

**Affiliations:** 1 State Key Laboratory of Quantum Optics and Quantum Optics Devices, Institute of Laser Spectroscopy, Shanxi University, Taiyuan, Shanxi 030006 China; 2 Collaborative Innovation Center of Extreme Optics, Shanxi University, Taiyuan, Shanxi 030006 China

## Abstract

In this paper, we present a novel ultra-narrow linewidth fiber resonator formed by a tunable polarization maintaining (PM) *π*-phase-shifted fiber Bragg grating and a PM uniform fiber Bragg grating with a certain length of PM single mode fiber patch cable between them. Theoretical prediction shows that this resonator has ultra-narrow linewidth resonant peaks and is easy to realize impedance matching. We experimentally obtain 3 MHz narrow linewidth impedance matched resonant peak in a 7.3 m ultra-long passive fiber cavity. The impedance self-matching characteristic of this resonator also makes itself particularly suitable for use in ultra-sensitive sensors, ultra-narrow band rejection optical filters and fiber lasers applications.

## Introduction

Recently, narrow linewidth fiber Bragg gratings (FBGs) become of paramount importance in many applications such as Bragg reflectors^1, 2^, optical filters^3, 4^, and optical sensors^5–10^, because of its significant advantages of easy to integrate and electromagnetic interference immunity^11^. Researchers have made a lot of efforts to get FBGs with narrow linewidth because narrow linewidth FBG can improve the system performance and the sensing sensitivity. A high resolution uniform FBG was made by fabricating a strong coupled FBG^12^, which can achieve ~2 pm (250 MHz) wavelength resolution. By using a special structure FBG, *π*-phase-shifted FBG, Painchaud *et al*. have attained ~0.5 pm (60 MHz) wavelength resolution^13^. Recently, cavity enhanced methodology has been realized by fabricating a FBG based ring resonator^14, 15^, or more often, by fabricating a fiber based Fabry-Perot interferometer^16–19^, where it is easy to obtain a high wavelength resolution cavity (~1 pm, 125 MHz for a 12 mm long cavity typically). In cavity enhanced methodology, an improvement of wavelength resolution can be achieved by increasing the cavity finesse or the cavity length. However, the reflectivity of FBG units, which is typically around 99%^20^, limits the further refinement of the finesse. Furthermore, for the traditional uniform Bragg grating based fiber cavity, the non-negligible loss in resonant cavity limits its cavity length because the increasing of cavity loss along with the lengthen of the cavity length increases the difficulty of impedance matching of cavity, where a cavity is impedance matched when the transmission of first mirror *t*_1_, the loss of cavity in a round trip of field propagation *l* and the transmission of second mirror *t*_2_ meet the condition *t*_1_ = *t*_2_ + *l* ^21^. Especially, the amplitude of cavity mode and the signal-to-noise ratio of signal will be drastically reduced without the impedance matching^22, 23^.

In this paper, we put forward an impedance self-matching ultra-narrow linewidth fiber resonator based on tunable *π*-phase-shifted FBG. Benefited from its impedance self-matching characteristic, and cavity enhanced effect, linewidth of impedance matched cavity mode of this resonator is down to 3 MHz, which to the best of our knowledge, is the narrowest linewidth in passive fiber based resonators.

## Fiber resonator based on tunable *π*-phase-shifted FBG

The configuration of fiber resonator is composed of a tunable polarization maintaining (PM) *π*-phase-shifted fiber Bragg grating (PM-PSFBG) and a PM uniform fiber Bragg grating (PM-FBG) with a certain length of PM single mode fiber patch cable (PM-FIBER) between them. It is important for a polarization disturbance immune operation that the whole structure is fabricated by a polarization maintaining process, and the input light has a linear state of polarization aligned to the slow axis of PM fiber. We refer to this structure as PSFBG-F-FBG resonator and to traditional fiber resonator formed by two PM uniform Bragg gratings as FBG-F-FBG resonator. Compared with FBG-F-FBG resonator, the PSFBG-based impedance self-matching characteristic makes PSFBG-F-FBG resonator easy to realize impedance matched condition, with which allows a longer cavity length, and then improves the wavelength resolution of this resonator.

**Figure 1:**
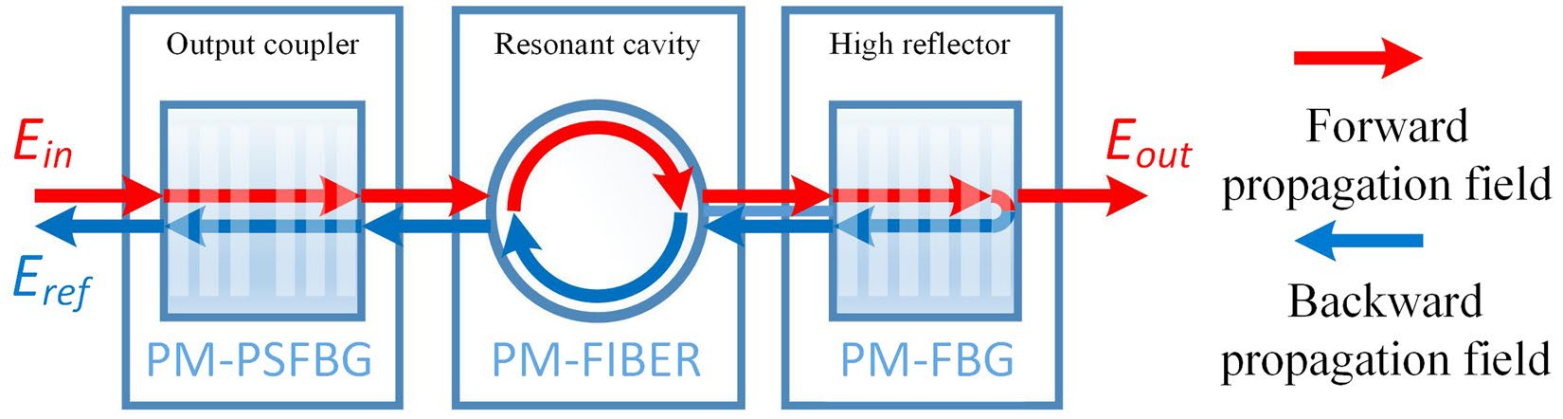
Sketch of PSFBG-F-FBG configuration. The red arrows and blue arrows indicate two counter propagating waves respectively. *E*_*in*_ stands for the input light field, *E*_*ref*_ for reflected light field, and *E*_*out*_ for transmitted light field.

The PSFBG-F-FBG configuration (see Fig. 1) is described as an optical cavity, which contains a PM *π*-phase-shifted Bragg grating as its output couple mirror, a PM single-mode fiber as its resonant cavity, and a PM uniform Bragg grating as its high reflectivity mirror. As fundamental matrix theory describes^24–27^, for a non-uniform structure contains *n* uniform sections, the propagation through each uniform section *i* is described by a matrix *F*_*i*_ defined such that

1 $$[\begin{array}{c}{E}_{i}^{+}\\ {E}_{i}^{-}\end{array}]={{\boldsymbol{F}}}_{i}[\begin{array}{c}{E}_{i-1}^{+}\\ {E}_{i-1}^{-}\end{array}],$$

where ${E}_{i}^{+}$ is the forward propagation light field and ${E}_{i}^{-}$ is the backward propagation light field after section *i* respectively. Then the fundamental matrix of total non-uniform structure will be

2 $${\boldsymbol{F}}=[\begin{array}{cc}{M}_{11} & {M}_{12}\\ {M}_{21} & {M}_{22}\end{array}]=\prod _{i=1}^{n}{{\boldsymbol{F}}}_{{\boldsymbol{i}}}.$$

The amplitude transmission and reflection coefficients of total structure can be obtained from the fundamental matrix elements, i.e. *t* = 1/*M*_22_ and *r* = −*M*_21_/*M*_22_, respectively. In PSFBG-F-FBG resonator, the total fundamental matrix *F*_*T*_, is given by

3 $${{\boldsymbol{F}}}_{{\boldsymbol{T}}}={{\boldsymbol{F}}}_{FBG}{{\boldsymbol{F}}}_{FIBER}{{\boldsymbol{F}}}_{PSFBG}.$$

For the intermediate item, if only considering the loss of optical fiber, the fundamental matrix of the fiber with fixed length *L* can be described as

4 $${{\boldsymbol{F}}}_{FIBER}=[\begin{array}{cc}{e}^{i\beta L}{10}^{-\frac{\alpha L+{\alpha }_{0}}{20}} & 0\\ 0 & {e}^{-i\beta L}{10}^{\frac{\alpha L+{\alpha }_{0}}{20}}\end{array}],$$

where *β* = 2*πn*_*eff*_/*λ* is the mode propagation constant (*λ* is the laser wavelength in vacuum, *n*_*eff*_ is the effective refractive index of optical fiber), *α* is the loss of optical fiber per meter, and *α*_0_ includes the splice loss of fiber and the insertion loss of fiber connectors. Both *α* and *α*_0_ are absolute values of loss in decibel. Assuming two grating units in PSFBG-F-FBG resonator are identical except that first grating has a *π*-phase-shifted in its center, and both of them are lossless and non-absorbing. The fundamental matrix of uniform grating is

5 $${{\boldsymbol{F}}}_{FBG}=[\begin{array}{cc}\frac{1}{{t}_{FBG}^{\ast }} & -\frac{{r}_{FBG}^{\ast }}{{t}_{FBG}^{\ast }}\\ -\frac{{r}_{FBG}}{{t}_{FBG}} & \frac{1}{{t}_{FBG}}\end{array}],$$

where

6 $${t}_{FBG}=\frac{{e}^{-2il{\rm{\Delta }}\beta }\gamma }{\gamma \,\cosh (2l\gamma )-i{\rm{\Delta }}\beta \,\sinh (2l\gamma )},$$

and

7 $${r}_{FBG}=\frac{{\rm{\Omega }}}{{\rm{\Delta }}\beta +i\gamma \,\coth (2l\gamma )},$$

are the amplitude transmission and reflection coefficients of uniform grating respectively. The fundamental matrix of *π*-phase-shifted grating is

8 $${{\boldsymbol{F}}}_{PSFBG}=[\begin{array}{cc}\frac{1}{{t}_{PSFBG}^{\ast }} & -\frac{{r}_{PSFBG}^{\ast }}{{t}_{PSFBG}^{\ast }}\\ -\frac{{r}_{PSFBG}}{{t}_{PSFBG}} & \frac{1}{{t}_{PSFBG}}\end{array}],$$

where

9 $${t}_{PSFBG}=\frac{{e}^{-2il{\rm{\Delta }}\beta }{\gamma }^{2}}{{{\rm{\Omega }}}^{2}-{\rm{\Delta }}\beta [{\rm{\Delta }}\beta \,\cosh (2l\gamma )+i\gamma \,\sinh (2l\gamma )]},$$

and

10 $${r}_{PSFBG}=\frac{-\,2{\rm{\Delta }}\beta {\rm{\Omega }}{\sinh }^{2}(l\gamma )}{{{\rm{\Omega }}}^{2}-{\rm{\Delta }}\beta [{\rm{\Delta }}\beta \,\cosh (2l\gamma )+i\gamma \,\sinh (2l\gamma )]},$$

are the amplitude transmission and reflection coefficients of *π*-phase-shifted grating respectively. Where Δ*β* = 2*πn*_*eff*_/*λ* − 2*πn*_*eff*_/*λ*_*B*_ is the differential wave number which means the detuning between laser wavelength (*λ*) and Bragg wavelength of Bragg grating (*λ*_*B*_), Ω = *π*Δ*n*_0_Γ*v*/*λ* is the coupling coefficient which describes the coupling efficiency between two counter propagating waves, here Δ*n*_0_ is the refractive index modulation amplitude of Bragg grating, Γ is the confinement factor relative to the ratio of guided energy in the core of fiber, and *ν* is the fringe visibility of the index change. The amplitude transmission and reflection coefficients of grating are obtained by using the coupled mode theory for two counter propagating waves, as this is detailed in numerous articles^28–30^. Our notation and calculation method follow most closely that of Martinez and Ferdinand^29^, for their notation is concise and calculation method can simultaneously describe the reflections of uniform grating and phase-shifted grating. From Eqs (3–10), we yield the amplitude reflection coefficient of PSFBG-F-FBG resonator,

11 $$r=\frac{{e}^{2iL\beta }{r}_{FBG}{t}_{PSFBG}+{10}^{\frac{\alpha L+{\alpha }_{0}}{10}}{r}_{PSFBG}{t}_{PSFBG}^{\ast }}{{e}^{2iL\beta }{r}_{FBG}{t}_{PSFBG}{r}_{PSFBG}^{\ast }+{10}^{\frac{\alpha L+{\alpha }_{0}}{10}}{t}_{PSFBG}^{\ast }},$$

and the reflection rate of PSFBG-F-FBG resonator *R* equals to

12 $$R=r{r}^{\ast }\mathrm{.}$$

## Characteristics of Fabry-Perot configuration based on *π*-phase-shifted FBG

### Reflection spectrum of PSFBG-F-FBG resonator

**Figure 2:**
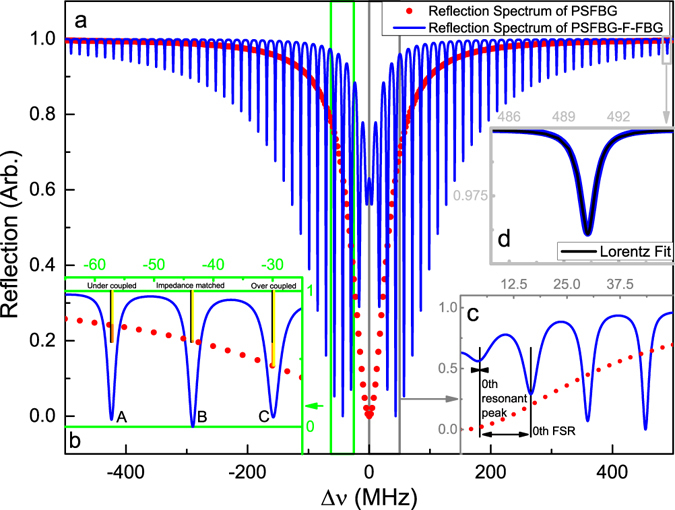
Theoretical spectrum reflection of 7.3 m long PSFBG-F-FBG resonator, plotted versus the frequency detuning Δ*v* = *C*/*λ* − *C*/*λ*_*B*_: (**a**) comparison of PSFBG-F-FBG resonator reflection spectrum (blue solid curve) and single phase-shifted grating reflection spectrum (red dot curve), (**b**) enlargement of green block in (**a**), with a description of impedance matching situations for points A, B and C, (**c**) enlargement of gray block in (**a**), with a description of resonant peak index number and free spectral range index number, (**d**) enlargement of light gray block in (**a**), with a Lorentz fit of single resonant peak.

Figure 2 shows the reflection spectrum of PSFBG-F-FBG resonator calculated from its reflection rate. Some grating parameters used for calculation are showed in Table 1. The effective refractive index *n*_*eff*_ and fiber loss *α* are 1.468 and 0.0002 dB/m respectively, which are the effective group index of refraction and loss of Corning SMF-28e+^®^ fiber at 1550 nm. The loss *α*_0_ contains connector insertion loss and splice loss. For ordinary FC/APC connectors, these connectors feature a low 0.25 dB connector-to-connector typical loss. The value of *α*_0_ estimates to be a sum of 0.5 dB connector insertion loss (0.25 dB for phase-shifted grating to fiber patch cable and 0.25 dB for fiber patch cable to uniform grating) and 0.5 dB splice loss. The value of grating refractive index modulation amplitude Δ*n*_0_ is 0.0017, length of grating unit *l* is 0.00128 m and Bragg wavelength *λ*_*B*_ is 1.55 *μ*m, which are the grating parameters we used in later experiment. An integer *N* is introduced to obtain a symmetrical reflection spectrum. As a characteristic of Fabry-Perot interferometer, the reflection spectrum is symmetrical when the length of fiber (resonant cavity length) is half-integer multiples of half of effective Bragg wavelength in fiber, as described in equation:

13 $$L=\frac{N}{2}\frac{{\lambda }_{B}}{2{n}_{eff}},$$

where *N* is 27630640, which is approximately equivalent to a fiber length of 7.3 m. Assuming the Bragg grating is a fully modulated grating, the grating fringe visibility of the index change *ν* = 1, and for a grating fabricated in a single mode fiber, the confinement factor Γ = 1.

**Table 1:** Parameters of fiber gratings in theoretical calculations and experiments.

	*n*_*eff*_	*α*	*α*_0_	Δ*n*_0_	*l*	*λ*_*B*_	*N*
value	1.468	0.0002	1	0.0017	0.00128	1.55	27630640
unit	—	dB/m	dB	—	m	μm	—

In Fig. 2(a), as a reference, the red curve is the reflection spectrum of a single *π*-phase-shifted Bragg grating around the Bragg wavelength. It shows that the spectrum of single *π*-phase-shifted grating has a narrow transmission peak at the position of the Bragg wavelength. The original transmission peak has a Lorentz profile and is about 66 MHz linewidth (FWHM). Due to the coordinate range limit, the reflection bandwidth (distance between the first zeros on either side of the Bragg wavelength in its reflection spectrum) of phase-shifted grating (2.2 nm) is not shown in the figure. Compared with the phase-shifted grating, the uniform grating only has a broadband reflection window, which has a 1.9 nm reflection bandwidth. The blue curve in Fig. 2 shows the reflection spectrum of PSFBG-F-FBG resonator. Note that there are several resonant peaks showing in PSFBG-F-FBG resonator spectrum because of Fabry-Perot interferometer structure.

### Impedance matching of PSFBG-F-FBG resonator

Figure 2(b) is an enlargement of green block in (a), and it shows the impedance matching characteristic of PSFBG-F-FBG resonator. The fiber resonator is impedance matched when it satisfies the impedance matching condition^31^, i.e. *T*_*PSFBG*_ = *T*_*FBG*_ + *l*_*FIBER*_, where ${T}_{PSFBG}={t}_{PSFBG}{t}_{PSFBG}^{\ast }$ and ${T}_{FBG}={t}_{FBG}{t}_{FBG}^{\ast }$ are the transmission rates of phase-shifted grating and uniform grating, respectively. *l*_*FIBER*_ is the total loss of fiber in a round trip of field propagation in percent, which could estimate from *α* and *α*_0_ by Eq. (14). The reflection rate of uniform grating near the Bragg wavelength is generally higher than 99%, so the transmission rate of this grating can be neglected, yield *T*_*FBG*_ ≈ 0. As a consequence, when the transmission rate of phase-shifted grating equals the loss of fiber, the cavity will have an impedance matched cavity mode.

14 $${l}_{FIBER}=1-{10}^{-2\ast \frac{\alpha L+{\alpha }_{0}}{10}}.$$

In Fig. 2(b), points A, B and C are three kinds of cavity modes with different impedance matching situations, which are caused by the variation of phase-shifted grating transmission rate. The black vertical bars indicate the loss in fiber, which are calculated to be 36.93% from Eq. (14) with parameters listed in Table 1. The yellow vertical bars indicate the transmission rate of phase-shifted grating and the orange vertical bars indicate the existing of reflection filed. The fiber loss remains unchanged at points A, B and C. For point A, the transmission rate of phase-shifted grating is 25.03%, i.e. *T*_*PSFBG*_ < *l*_*FIBER*_, which means this cavity mode is in a significant under-coupled situation. For point C the transmission rate of phase-shifted grating is 54.87%, i.e. *T*_*PSFBG*_ < *l*_*FIBER*_, and this cavity mode is in a significant over-coupled situation. At point B the transmission rate of phase-shifted grating is 36.42%, i.e. almost satisfies the condition *T*_*PSFBG*_ < *l*_*FIBER*_, and this cavity mode is in an impedance matched situation, thus has almost zero reflection field.

**Figure 3:**
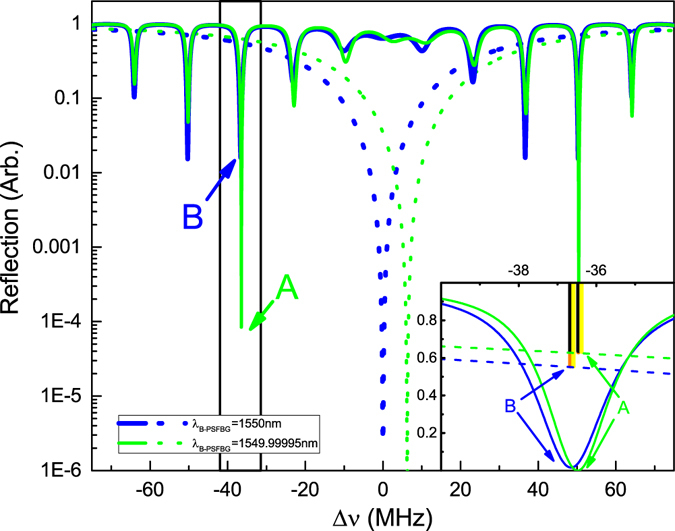
Theoretical spectrum reflection of 7.3 m long PSFBG-FPI-FBG resonator, plotted versus the frequency detuning Δ*v* = *C*/*λ* − *C*/*λ*_*B*_. The blue solid line and blue dot line are the reflection spectrum of PSFBG-F-FBG resonator and single PSFBG with a Bragg wavelength of 1550 nm, respectively. The green solid line and green dot line are the reflection spectrum of PSFBG-F-FBG resonator and single PSFBG with a Bragg wavelength of 1549.99995 nm, respectively. Inset: Detail description of impedance matching situation of points A and B.

However, in most cases, the practical cavity length and grating parameters are not very appropriate thus cannot realize an ideal impedance matching. A typical example is shown to as the blue curve in Fig. 3. The curve is simulated with almost the same parameters in Table 1, while the integer number *N* changes to 27630641. At the best impedance matching point B, this configuration has about 1.5% reflected light power. But in the PSFBG-F-FBG resonator, the impedance matching situation can be improved by deploying a tunable phase-shifted grating. E.g., when the Bragg wavelength of phase-shifted grating has about 0.05 pm blue shift, the impedance matching of point B will be significantly improved, and the reflection light power decreases to 10^−4^ magnitudes, which is shown to be point A at green curve in Fig. 3. Since the blue shift of Bragg wavelength changes the transmission rate of phase-shifted grating (As a reference, the reflection spectrum of phase-shifted grating with a Bragg wavelength of 1550 nm is shown as the blue dot curve and the reflection spectrum of phase-shifted grating with a Bragg wavelength of 1549.99995 nm is shown as the green dot curve.) at the resonant frequency, which makes the transmission rate of phase-shifted grating equal to the fiber loss, thus realizes an impedance matched cavity mode. The tunable phase-shifted Bragg grating can be realized either by tuning the temperature of grating unit (for the grating with a Bragg wavelength of 1550 nm, about 0.004 °C temperature reduced will cause 0.05 pm blue shift of Bragg wavelength), or by supplying an extra strain to grating unit (for a grating with a Bragg wavelength of 1550 nm, about −0.035 *με* strain applied will cause 0.05 pm blue shift of Bragg wavelength, since the typical normalized strain and temperature response of PSFBG are 0.78 × 10^−6^ *με* ^−1^ and 6.67 × 10^−6^ °C^−1^ respectively^11^). A refined description of impedance matching situations of point A and point B by three color vertical bars defined before is shown in the inset in Fig. 3.

### Linewidth of PSFBG-F-FBG resonator

**Figure 4:**
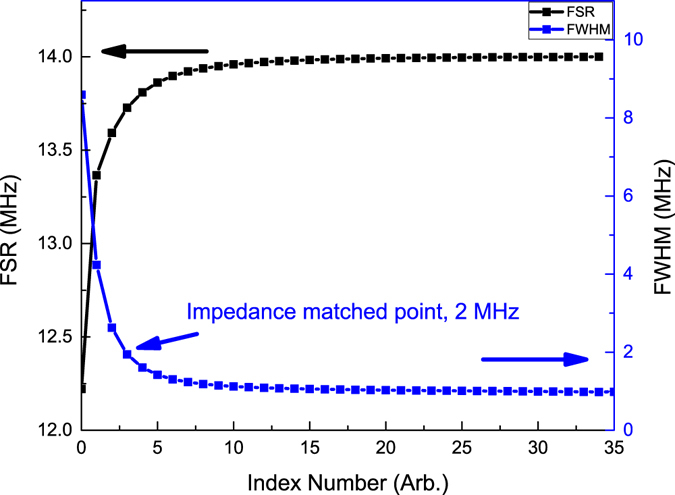
Theoretical linewidth (FWHM, blue line) and FSR (black line) of 7.3 m long PSFBG-F-FBG resonator, plotted versus the index number.

Figure 4 shows the linewidth (FWHM) of resonant peaks versus index number of resonant peaks and the free spectral range (FSR) versus FSR index number. The definitions of index number of resonant peaks and index number of FSR are shown in Fig. 2(c), which is an enlargement of gray box in Fig. 2(a). Along the one side of the Bragg wavelength, there are several local minima, and the local minimum which is the nearest to the Bragg wavelength has the index number zero and then the second from the Bragg wavelength has the index number one, and so forth. The free spectrum range is defined as the distance between two local minima, and the FSR between resonant peak with index number one and resonant peak with index number zero has a FSR index number zero, etc. For the symmetrical (or nearly symmetrical) reflection spectrum, the resonant peak index and FSR index along the other side of the Bragg wavelength can be defined by the same method. The FWHM of resonant peaks are obtained by Lorentz fits of each resonant peak, e.g., a single Lorentz fit of resonant peak is showed in Fig. 2(d), which is an enlargement of light gray block in Fig. 2(a).

As a consequence, the FWHM of resonant peak decreases when the index number increases. In the configuration with parameters listed in Table 1, this resonator has about 2 MHz FWHM at the impedance matched point, and the linewidth could reduce to 1 MHz along with the increase of index number. The FSR of this resonator has an opposite behaviour, and it will increase when the index number increases. This means that the resonator doesn’t have a very high finesse at the impedance matched point, but has a high finesse when the detuning between the laser frequency and the Bragg wavelength is large. However, the amplitude of resonant peak also decreases when the detuning increases, and the impedance matched point is the optimal balanced point considering the linewidth and amplitude of resonant peak.

The shrinking of free spectral range is mainly caused by a strong dispersion effect of phase-shifted grating. For the cavity consisting of two FBGs, the cavity FSR is mainly determined by the group delay time of light reflected between them. The property of the cavity can be described in terms of the effective cavity length, in which the FBGs’ effective lengths should be accounted for. A simple analytical formula for calculation of the effective length of the uniform FBG in a Fabry-Perot fiber cavity has been presented by Barmenkov etc.^32^. In this way we can obtain the effective length of the phase-shifted grating in PSFBG-F-FBG resonator, which is obtained from the phase of reflection coefficient of grating, and describes by:

15 $${L}_{eff}=-\,\frac{{\lambda }^{2}}{4\pi {n}_{eff}}\frac{d\varphi }{d\lambda },$$

where $\varphi ={\tan }^{-1}(\Im {r}_{PSFBG}/\Re {r}_{PSFBG})$ is the phase of reflection coefficient of phase-shifted grating. Unlike the effective cavity length of uniform grating given by Barmenkov etc., the effective length of phase-shifted grating can be significantly larger than its unit length (When the laser frequency is resonant to the Bragg wavelength of phase-shifted grating, the effective length of phase-shifted grating has a simple form as shown as Eq. (16), and for parameters listed in Table 1, the effective length of phase-shifted grating will be approximately 0.5 m, which is nearly 400 times as its original unit length.), which will increase the resonator cavity length, and finally induce the shrinking of the free spectral range of cavity.

16 $${L}_{eff}=\frac{{\lambda }_{B}\,\sinh (\frac{2l\pi {\rm{\Delta }}{n}_{0}{\rm{\Gamma }}\nu }{{\lambda }_{B}})}{2\pi {\rm{\Delta }}{n}_{0}{\rm{\Gamma }}\nu }.$$

**Figure 5:**
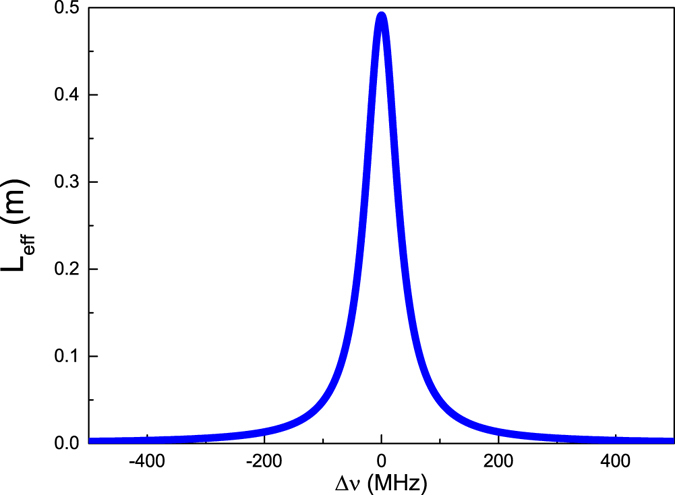
Effective cavity length of phase-shifted grating in PSFBG-F-FBG resonator, plotted versus the frequency detuning Δ*v* = *C*/*λ* − *C*/*λ*_*B*_.

Figure 5 shows the effective cavity length versus detuning between laser frequency and the Bragg wavelength of phase-shifted grating, the effective length of phase-shifted grating will increase when detuning decreases, and the shrinking of FSR is due to the integral effect of this property.

## Experiment setup

**Figure 6:**
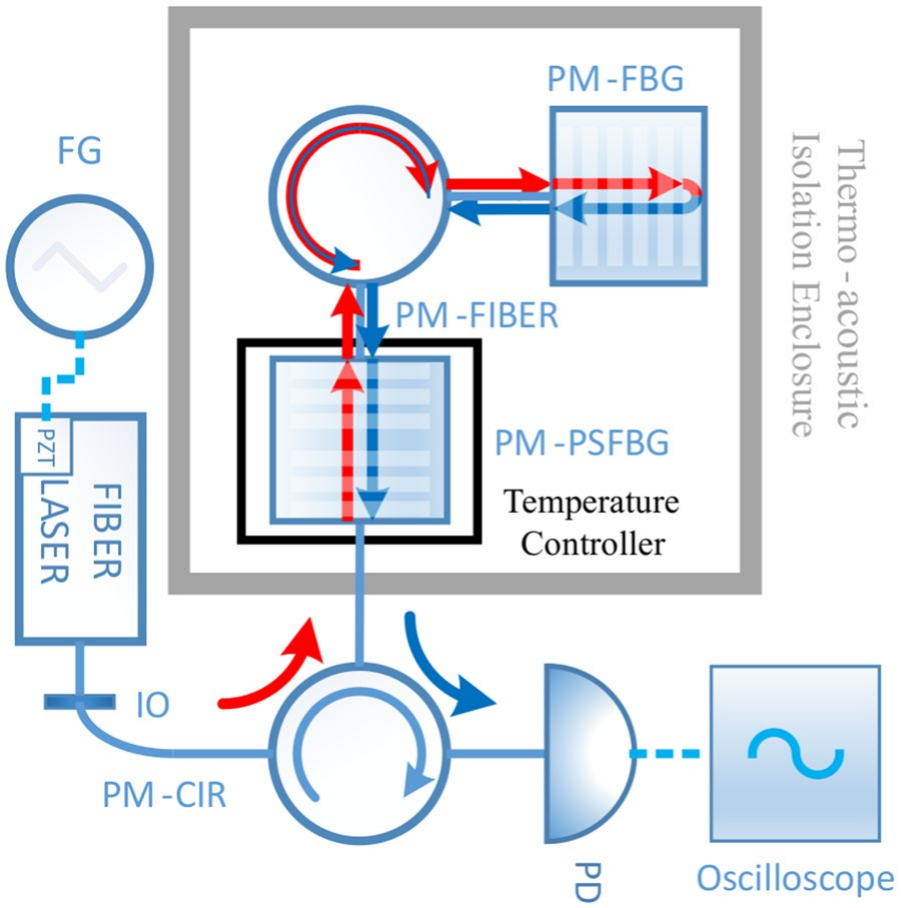
Sketch of the experimental setup. FG stands for function generator, IO for polarization maintaining isolation, PM-CIR for polarization maintaining circulator, PM-PSFBG for polarization maintaining phase-shifted fiber Bragg grating, PM-FIBER for polarization maintaining fiber, PM-FBG for polarization maintaining uniform fiber Bragg grating, and PD for photo detector. The PM-PSFBG is temperature-controlled by a temperature controller. The PSFBG-F-FBG resonator is thermo-acoustic isolated from environment by an isolation enclosure and vibration isolated by a flotation optical platform.

We experimentally studied the reflection spectrum of PSFBG-F-FBG resonator. The experiment setup is showed in Fig. 6, the PSFBG-F-FBG resonator was formed by a polarization maintaining phase-shifted grating (TeraXion) and a polarization maintaining uniform grating (TeraXion) which are linked by a polarization maintaining fiber patch cable. Both of the gratings used in experiment have the same grating parameters listed in Table 1. The reflection light field was guided by a polarization maintaining optical circulator (Thorlabs, CIR1550PM) to a broad bandwidth InGaAs photo detector (New Focus, 1611) and the output current of detector was monitored by a broad bandwidth mixed signal oscilloscope (YOKOGAWA, DLM2000). The cavity reflection was obtained by using a tunable single frequency fiber laser (Orbits Lightwave, ETH-1550) with an effective linewidth of 250 Hz calculated from FM noise spectrum. The light was output through a PM fiber from laser with 27 dB polarization extinction. Fully PM fiber components and the high polarization extinction of laser source are helpful to against polarization fading. The polarization fading will reduce the visibility of resonant peaks, thus degenerate the resonator performance. It is worth to indicate that some new kinds of highly birefringent FBG^33^ could further improve the resonator performance. The PSFBG-F-FBG resonator was placed in a thermo-acoustic isolation enclosure to reduce the fluctuation of laser polarization and cavity length caused by ambient temperature or sound pressure. In order to temperature-tuning the phase-shifted FBG, we directly fixed the PSFBG unit to a thermoelectric cooling (TEC) by thermal conductive tape, and wrapped the TEC by flexible insulation material to isolate the influence of ambient temperature and sound pressure. An analog voltage was inputted to the temperature controller LFI-3751 (Wavelength Electronics) to fine adjust the control temperature in order to obtain 0.01 °C temperature step. The temperature fluctuation when the system achieved thermal equilibrium is about 0.002 °C.

**Figure 7:**
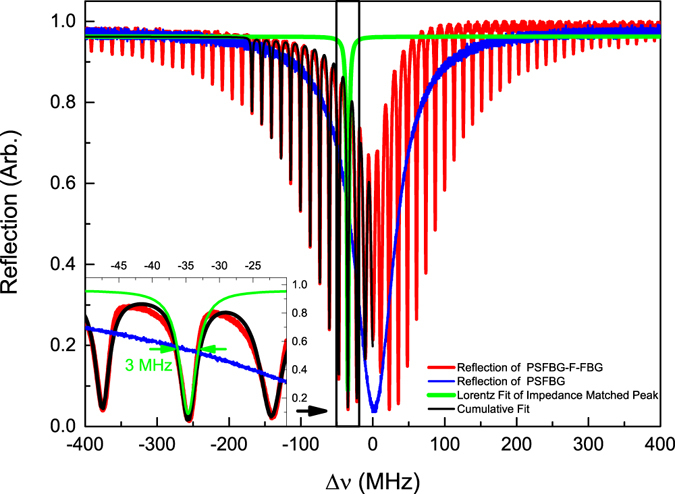
Experimental spectrum reflection of 7.3 m long PSFBG-F-FBG resonator, plotted versus the frequency detuning Δ*v* = *C*/*λ* − *C*/*λ*_*B*_. The red line is the reflection spectrum of PSFBG-F-FBG resonator, the blue line is the reflection spectrum of single PSFBG, the green line is the Lorentz fit of impedance matched resonant peak, and the black line is the cumulative fit of experiment data. Inset: Detail description of reflection spectrum of PSFBG-F-FBG resonator at the impedance matching peak.

Figure 7 shows the experimental reflection spectrum of PSFBG-F-FBG resonator, the blue curve is the reflection spectrum of a single phase-shifted grating, which has a Lorentz profile with 68 MHz linewidth, and the red curve is the reflection spectrum of PSFBG-F-FBG resonator. In Fig. 7, the experimental spectrum generally agrees well with the theoretical spectrum, although the spectrum is asymmetry because the cavity length is difficult to ascertain in the experiment to satisfy the condition given by Eq. (13). According to the Lorentz fit of impedance matched resonant peak in reflection spectrum of PSFBG-F-FBG resonator (green line), the linewidth of impedance matched resonant peak is about 3 MHz, which is benefited from the cavity enhanced effect, long cavity length (about 7.3 m in this figure) and impedance self-matching characteristic mentioned before. From the inset figure in Fig. 7, it is find that the peak of single Lorentz fit curve is lower than the experiment result, which is because the total reflection spectrum is a sum effect of multi Lorentz profile resonant peaks. A cumulative fit of experiment data shown in black data agrees well with the experiment spectrum at most positions, although the fit error is large around the Bragg wavelength, which is caused by the change of fit base line due to the asymmetry of maximum reflection light power in reflection spectrum.

**Figure 8:**
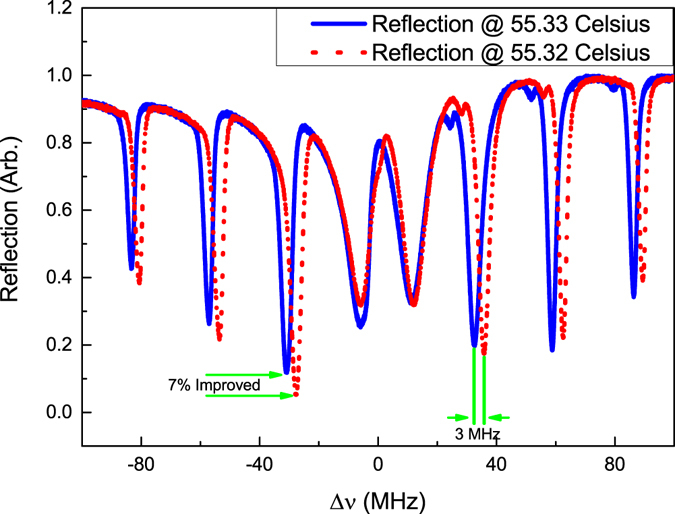
Experimental example of using a tunable PSFBG to improve the impedance matching of resonant peak. Here the Bragg wavelength of PSFBG is tuned by changing its temperature. The blue curve is the reflection spectrum of 3.4 m long PSFBG-F-FBG resonator when temperature of PSFBG is 55.33 °C, and the red dot curve is the reflection spectrum of the same PSFBG-F-FBG resonator when temperature of PSFBG is 55.32 °C.

An experimental example of improvement of PSFBG-F-FBG resonator’s impedance matching is showed in Fig. 8. Here the cavity length of PSFBG-F-FBG resonator was changed to 3.4 m, in order to show the influence of different cavity length on the spectrum. At first the temperature of PSFBG was set to 55.33 °C, and as the experiment result shows, the minimal reflection light power was about 12%. The impedance matching situation can be improved by changing the Bragg wavelength of PSFBG, which was realized by changing the temperature of PSFBG unit in this experiment. When the temperature around the PSFBG was changed to 55.32 °C, the reflection light power reduced to 5%, which had about 7% improvement relative to that of 55.33 °C. Compared with the theoretical prediction, the experimental improvement result is limited by the tuning accuracy of PSFBG’s Bragg wavelength, relating to the accuracy of temperature controller in our experiment. The frequency/temperature response coefficient of PSFBG (blue line) is about 1.25 GHz/°C (equals to a normalized temperature response of 6.46 × 10^−6^ °C^−1^, almost the same with the value mentioned before), and a 0.01 °C temperature change equals 12.5 MHz Bragg frequency shift (or 0.1 pm Bragg wavelength shift). However the reflection peak of PSFBG-F-FBG resonator only showed a 3 MHz shift. The resonant frequencies of this resonator directly depend on the effective cavity length of resonator, which is influenced by the effective length of PSFBG. The effective length of PSFBG is a function of detuning between laser frequency and the Bragg frequency of PSFBG, which is showed in Fig. 5. As a consequence, the blue shift of PSFBG Bragg wavelength will increase the frequency detuning at resonant frequencies, then decrease the effective cavity length of PSFBG itself, and further decrease the total effective cavity length of PSFBG-F-FBG resonator. The decrease of total effective cavity length caused the blue shift of resonant peaks, which has a nonlinear relationship with the blue shift of PSFBG Bragg frequency.

**Figure 9:**
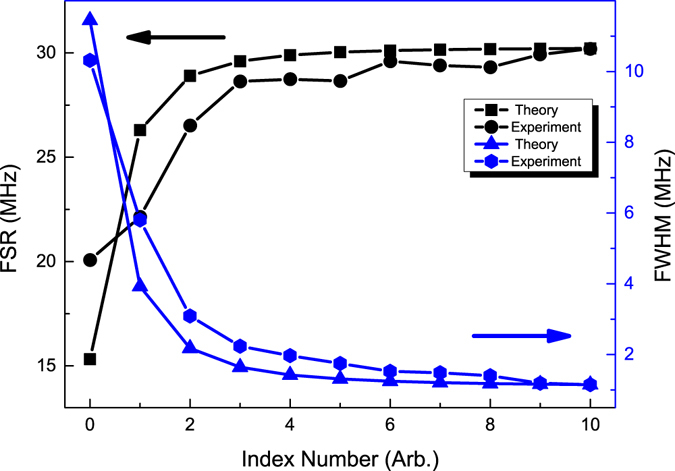
Experimental results and theoretical results of FSR and linewidth of a 3.4 m long PSFBG-F-FBG resonator, plotted versus the index number.

Figure 9 shows the experimental and theoretical FSR (black line with round points and black line with square points respectively) and linewidth (FWHM) (blue line with hexagonal points and blue line with triangular points respectively) of the PSFBG-F-FBG resonator with a cavity length of 3.4 m. The experimental results agree well with the theoretical predictions with a large index number where means a large frequency detuning between the laser frequency and the Bragg frequency of PSFBG, but have low confidence when the index number is small. This is because the grating parameters of experiment cannot be exactly the same as theoretical calculation, which will lead to an inappropriate estimate of dispersion effect of PSFBG. The FSR in experiment data at far detuning frequency is about 30 MHz and linewidth is about 1 MHz, which yields a finesse of 30. At the impedance matched frequency, the FSR is about 22 MHz and linewidth is about 6 MHz, which yields a finesse of 4. This implies that the highest finesse of this resonator with a given cavity length is not obtained at the impedance matched cavity mode. The optimal choice of cavity mode depends on the type of applications, and for conventional purpose the impedance matched cavity mode is an optimal balanced mode between the amplitude of cavity mode and the coherent linewidth. For the impedance matched cavity mode, the linewidth of 3.4 m long cavity is twice as the linewidth of 7.3 m long cavity. This implies that the linewidth of impedance matched resonant peak can be further improved by continuously increasing the cavity length, until the broadening effect on linewidth owing to the decreasing of cavity finesse, which depends on the transmission rate of PSFBG, suppresses the narrowing effect on linewidth attributed to the increasing of cavity length.

**Figure 10:**
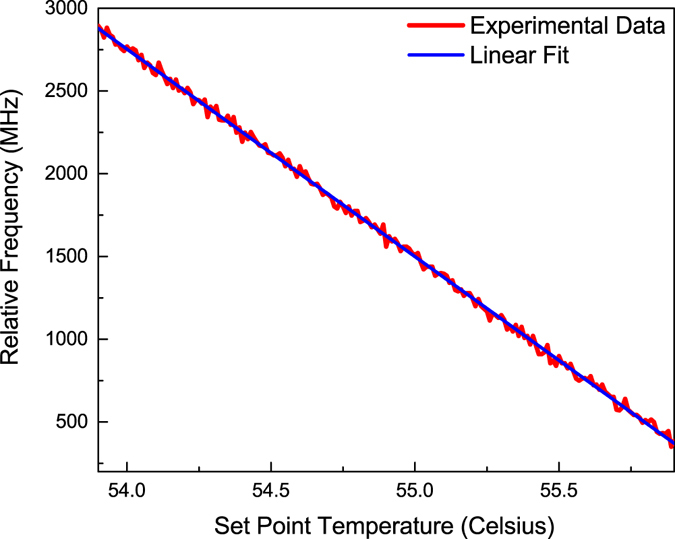
Optical response of PSFBG-F-FBG resonator, plotted versus the set point temperature.

Figure 10 shows the optical response of PSFBG-F-FBG sensor as a function of controlling temperature. In order to obtain its optical response to temperature, the PSFBG-F-FBG resonator was coiled into a 20 mm radius ring and putted into a 50 × 50 × 15 mm temperature controlled aluminum rectangle box. The probe laser is a diode laser with a polarization linearity >100:1 (DL PRO, Toptica), which was locked to a resonant peak of ultra-stable Fabry-Perot cavity by PDH technique. The output light of the stabilized laser was modulated by an EO phase modulator (EOSPACE) to generate second carriers. The drive signal of the phase modulator was generated by a voltage-control oscillator, to scan the frequency of second carriers to obtain the relative frequency between 3 MHz narrow linewidth resonant peak of PSFBG-F-FBG resonator and the laser carrier. For each data point, the control temperature of LFI-3751 was set and waited for 30 seconds until the system reached thermal equilibrium where the temperature fluctuation was less than 0.01 °C. According to the linear fit of experiment results in Fig. 10, the slope of frequency-temperature response of PSFBG-F-FBG sensor is about −1250 MHz/°C. To the 3 MHz narrow linewidth resonant peak, its temperature resolution can reach to 0.002 °C.

## Conclusion

A PSFBG-F-FBG resonator shows several ultra-narrow linewidth resonant peaks, because the cavity enhanced effect of long cavity length Fabry-Perot interferometer structure and the impedance self-matching property by use of a tunable PSFBG. The impedance matched resonant peak is an optimal balanced point between the amplitude of cavity mode and the coherent linewidth. By using this resonator, we demonstrated a 3 MHz narrow linewidth resonant peak with a 7.3 m long fiber resonator. This resonator can reach to 0.002 °C temperature resolution when used as a temperature sensor. This resolution can be further improved by increasing the cavity length with lower-loss fiber. The ultra-narrow linewidth fiber resonator can be also used a narrow band rejection optical filter, and Bragg mirror pairs in fiber lasers. Furthermore, the impedance self-matching optical cavity make itself suitable for cavity enhanced absorption spectroscopy and cavity ringdown spectroscopy applications.
